# A Case of a Paracardial Osteophyte Causing Atrial Compression

**DOI:** 10.1155/2016/4325830

**Published:** 2016-12-29

**Authors:** Stergios Tzikas, Konstantinos Triantafyllou, Christodoulos Papadopoulos, Vassilios Vassilikos

**Affiliations:** 3rd Department of Cardiology, Ippokrateio Hospital, Aristotle University of Thessaloniki, Thessaloniki, Greece

## Abstract

Osteophytes are pointed or beaked osseous outgrowths at the margins of articular surfaces that are often associated with degenerative changes of articular cartilage. They are the most common aspect of osteoarthritis and they infrequently cause symptoms by compression of the adjacent anatomic structures, such as nerves, vessels, bronchi, and esophagus. We present here a rare case of a patient with a left atrial deformation by a large osteophyte.

## 1. Introduction

Dyspnea is the key symptom of heart failure, which accounts for 1 in 9 deaths in the United States in the year 2013 [1]. However various extracardiac conditions can also lead to dyspnea, complicating the differential diagnosis. The structural deformation of cardiac chambers and of the pulmonary veins is among rare cases of cardiac dyspnea [2].

The shape of the cardiac chambers may be shown deformed usually by cardiac masses, tumors, thrombi, and cysts. Osteophytes are pointed or beaked osseous outgrowths at the margins of articular surfaces that are often associated with degenerative changes of articular cartilage. They are the most common aspect of osteoarthritis and they infrequently cause symptoms by compression of the adjacent anatomic structures, such as nerves, vessels, bronchi, and esophagus. We present a rare case of a patient with dyspnea and left atrial deformation by a large osteophyte.

## 2. Case Presentation

A 79-year-old male presented to our out-patient clinic with dyspnea at mild exercise (New York Heart Association classification of II) and back pain for the previous 3 months. His medical history was significant for arterial hypertension, chronic atrial fibrillation, mild normochromic anemia of unknown cause, and osteoarthritis.

The clinical examination revealed dominant jugular veins and a systolic murmur.

**Figure 1:**
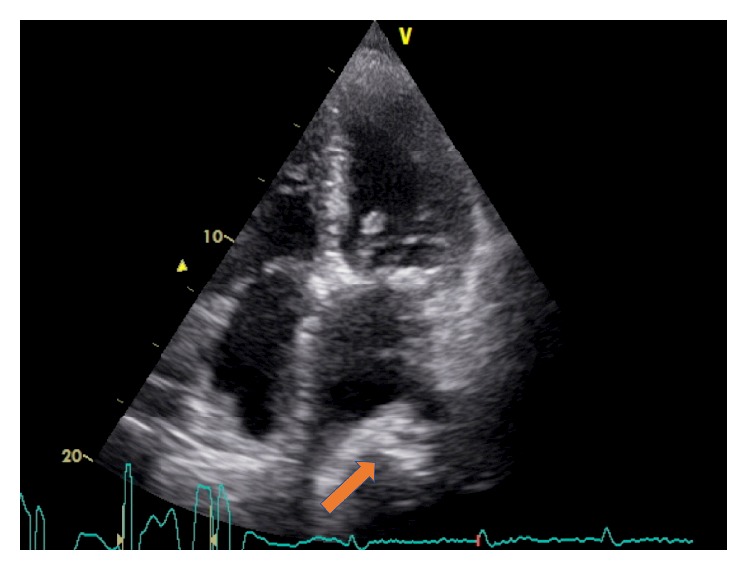
Echocardiography (four-chamber view) of the heart depicting a mass compressing the left atrium.

A transthoracic echocardiogram (Figure 1) was performed and revealed a normal systolic function of the left ventricle. The left atrium was severely dilated (52 × 58 mm, 40 mL/m^2^) and extrinsically deformed by a mass of unknown origin. Further echocardiographic findings included a heavily calcified mitral annulus with moderate mitral stenosis (mean pressure gradient: 6 mmHg, mitral valve area 1.7 cm^2^) and mild mitral regurgitation. In addition, the ascending aorta and the right atrium were mildly dilated, a mild tricuspid regurgitation appeared, and the right ventricular systolic pressure was estimated at 48 mmHg.

The aforementioned findings were confirmed by a subsequent transesophageal echocardiogram, as well as an apparent indentation in the posterior left atrial wall, while the pulmonary venous flow appeared unaffected.

**Figure 2:**
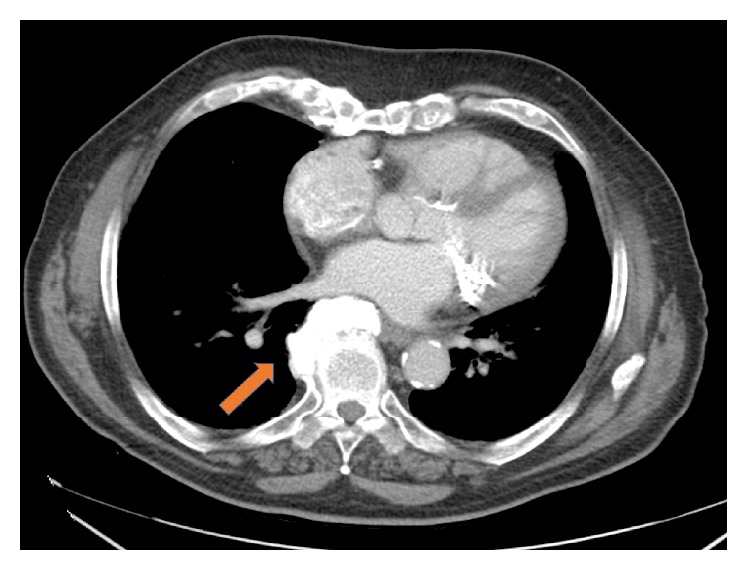
Computer tomography of the chest showing (arrow) the osteophyte of the left atrium.

**Figure 3:**
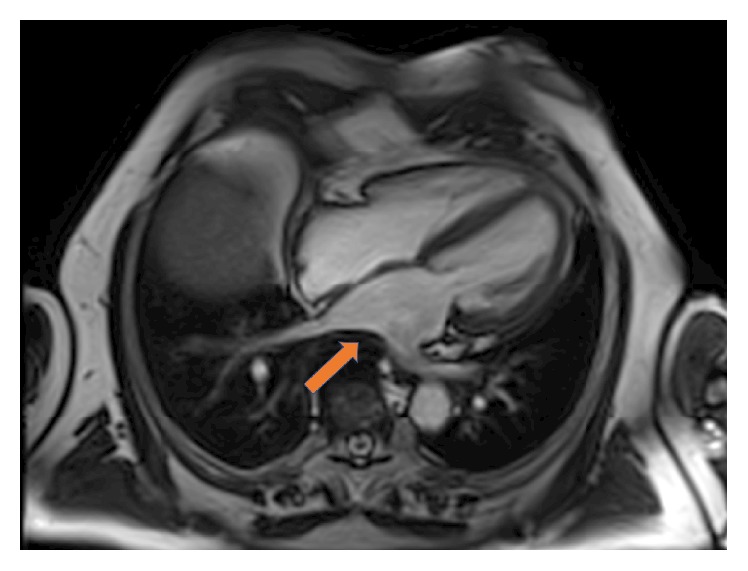
Magnetic resonance image of the heart showing the osteophyte protruding into the left atrium.

Chest Computer Tomography (CT) was performed (Figure 2) in order to further investigate the origin of the left atrial compression. An osteophyte was arising at the level of the seventh and eighth thoracic (T7-T8) vertebrae, which was large enough to protrude into the posterior wall of the left atrium. These findings were confirmed using magnetic resonance imaging (Figure 3).

The diagnostic evaluation contributed to the final diagnosis of heart failure due to mitral valve degeneration. Pulmonary hypertension was attributed to the presence of moderate mitral stenosis. The patient was prescribed diuretics, which led to gradual improvement of his clinical status.

## 3. Discussion

Osteophytes are osseous outgrowths located at the margins of articular surfaces. They are usually diagnosed incidentally during imaging examinations in elderly individuals, as they are mostly asymptomatic. However, several complications have been reported due to the presence of vertebral osteophytes. The most frequent complications are myelopathy and radiculopathy which occur because of mechanical compression of the vertebral canal [3, 4] and dysphagia, caused by mechanical compression of the esophagus [5–15]. Other rarer complications may result from external compression of the trachea [16, 17], the bronchi [18], the adjacent arteries [19–22], and nerves [23, 24]. Furthermore, chronic throat symptoms [25], back pain [26], Brown-Sequard syndrome [27], Horner syndrome [28], intracranial hypotension [29, 30], chronic obstructive pneumonia [31], traumatic thoracic aortic rupture [32], esophageal perforation [33], and acute urinary retention [34] have been described as osteophytic complications. As far as heart complications are concerned, a traumatic heart perforation [35] and two cases of left atrial deformation by large osteophytes [26, 36] have been so far reported.

In our case a large osteophyte compressed the left atrium. The transthoracic echocardiogram led to the suspicion of pulmonary veins compression. This hypothesis could be rejected by the means of transesophageal echocardiography. Transesophageal echocardiography is a useful tool for pulmonary vein investigation, although there are no validated criteria for the definition of pulmonary vein (PV) stenosis. It seems that an increased maximum PV Doppler flow velocity (>1.1 m/s) combined with color Doppler turbulence may be a reliable index [37, 38].

Vertebral osteophytes are common in the general population but very rarely protrude into the left atrium. This condition is rare, with fewer than 5 previously reported cases. Our case is similar to previously reported, except that we believe this is the first reported case with suspicion of pulmonary vein stenosis.
